# Supplementary material for: A bioinspired multilegged soft millirobot that functions in both dry and wet conditions
Source: Nat Commun. 2018 Sep 26;9:3944. doi: 10.1038/s41467-018-06491-9 (PMC6158235; doi:10.1038/s41467-018-06491-9)
Supplement: Supplementary file 1 — Supplementary information [file 41467_2018_6491_MOESM1_ESM.pdf]

Supplementary Information for

A bioinspired multilegged soft millirobot that functions in both dry and wet conditions

Haojian Lu[1†], Mei Zhang[1†], Yuanyuan Yang[1], Qiang Huang[2], Toshio Fukuda[2], Zuankai Wang[1,3,*], Yajing Shen[1,3,4,*]

[1]Department of Mechanical and Biomedical Engineering, City University of Hong Kong, Hong Kong, China

[2]Beijing Institute of Technology, Beijing, China

[3]Shenzhen Research Institute of City University of Hong Kong, Shenzhen 518057, China

[4]Department of Biomedical Engineering, City University of Hong Kong, Hong Kong, China

[†]These authors contributed equally to this work.

*Correspondence to: Zuankai Wang (zuanwang@cityu.edu.hk); Yajing Shen (yajishen@cityu.edu.hk)

This PDF file includes:

Supplementary Figures

Supplementary Methods

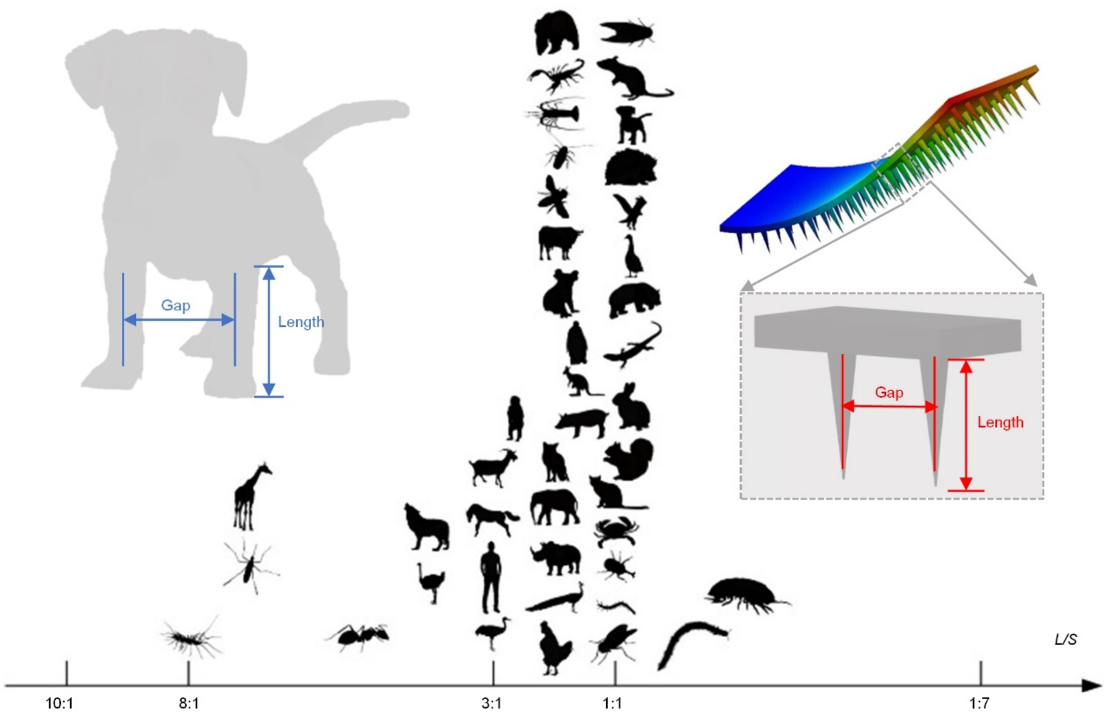

Supplementary Figure 1 | Dimensionless analysis of the length to spacing ratio. Here, the spacing is defined as the distance between the feet/legs in the same row. Furthermore, the "gap ratio (*L/S*)" is defined as the length of the tapered feet (or leg) *L* relative to the spacing between two tapered feet (or leg) *S* in the same row legs. On the basis of extensive review of structural geometry of hundreds of ground animals, we find that the length of their legs (*L*) is normally 1~2 times larger than that of their foot-to-foot spacing (*S*). We found that the *L/S* of most legged animals ranges between 1 and 2. We hypothesis that it's a compromise between efficient locomotion and body supporting, since a large *L/S* is preferential for locomotion, but inferior to body supporting; to contrary, a small *L/S* is preferential for body supporting, but limited in locomotion. Interestingly, for animals with soft legs or feet such as starfish, centipede and pillworm, the *L/S* is typically approximately close to unity to make up the weakness of soft legs in body supporting. Thus, in our work, we chose $L/S \approx 1$ (see Supplementary Methods).

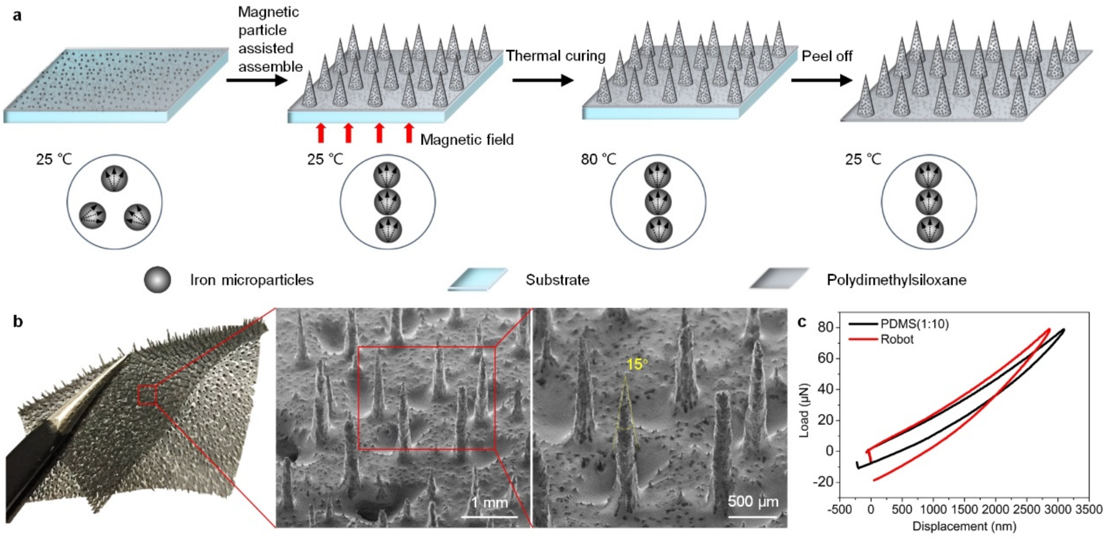

Supplementary Figure 2 | Multi-legged millirobot manufacture and measurement. a, Manufacture process of the multi-legged millirobot with flexible tapered feet (see Supplementary Methods). Mixture containing Polydimethylsiloxane (PDMS) pre-polymer and iron microparticles are first deposited on a polystyrene plate via spinning coating. Then, a magnet bar is put underneath to induce the formation of tapered feet under the guidance of magnetic field. After a solidification in a convection oven for thermal curing for 1 h at 80 °C, the as-fabricated film is peeled off from the plate and cut into the rectangular shape. **b,** SEM observation of robot. The thickness of body, foot length and foot-to-foot spacing of robot is ~150, ~650, and ~600 μm, respectively. The tip angle of the tapered feet is ~15°. **c,** Young's modulus measurement of robot. Since the Young Modulus of the as-fabricated soft body is measured to be ~2 MPa, we refer to our robot as soft multi-legged millirobot.

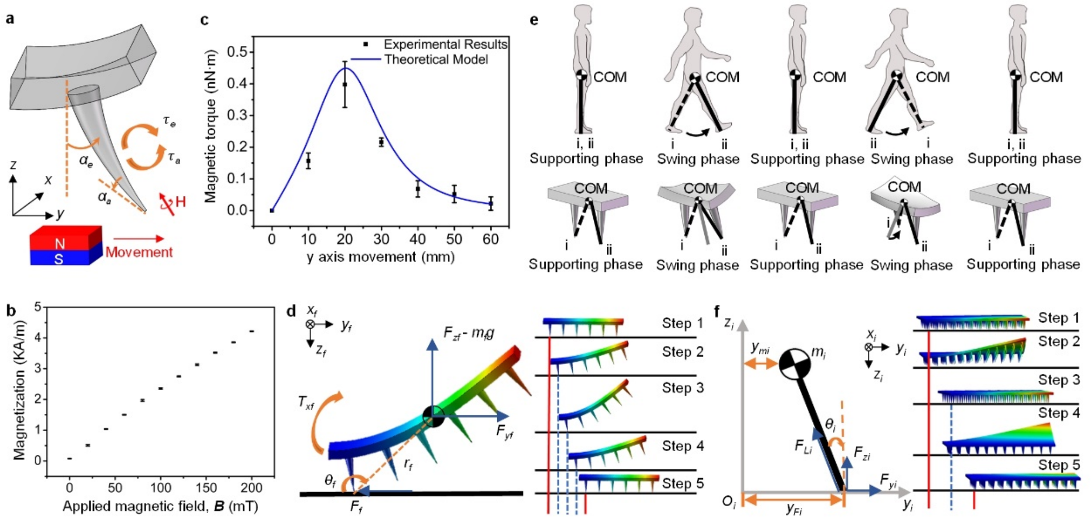

Supplementary Figure 3 | Magnetic mechanism analysis and two motion modes. a, Schematic drawing of the single tapered foot. The single tapered foot is treated as a whole unit, regardless of the inner microstructure of iron microparticles (see Supplementary Methods). **b,** The magnetization of the single tapered foot under different applied magnetic field strength. As the magnetic field strength applied at the tapered feet increase from 0 mT to 200 mT, the magnetization of the tapered feet increase from 0.08 kA/m to 4.22 kA/m. **c,** The magnetic torque of the single tapered foot. The theoretical value of magnetic torque applied to the tapered foot agrees well with the experimental results, which verifies the magnetic driven model. **d,** Discontinuous flap-wave mode model (see Supplementary Methods). To model the dynamics of the bioinspired millirobot with flexible tapered feet, we restrict modeling to a side-view of the robot in the *x*-*z* plane. The robot dynamic relations are developed with several parameters defined. **e,** Human-like robot locomotion. During human walking, the gait is performed as follows: 1. Two legs are standing on the ground (supporting phase), 2. Lift one leg off of the ground and use the leg in contact with the ground, push the body forward (swing phase), 3. Swing the lifted leg forward until it is in front of the body (swing phase), 4. Fall forward to allow the lifted leg to contact the ground (supporting phase). Repeat steps 1–4 for the other leg. **f,** Continuous inverted-pendulum mode model (see Supplementary Methods**).** Compared with human walking, the robot shows the similar gait steps, which can be analyzed mathematically via human's inverted pendulum locomotion model.

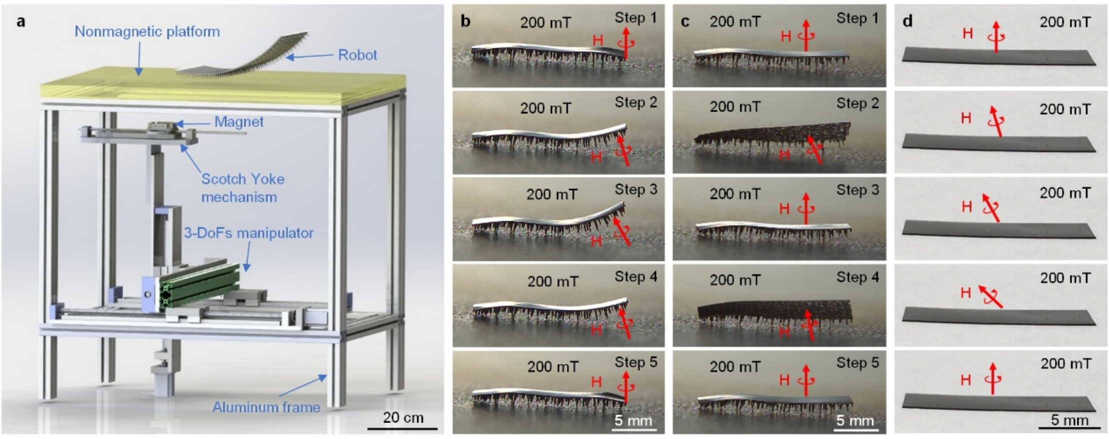

Supplementary Figure 4 | Manipulation system and robot motion comparison. a, Whole manipulation setup for robot actuation. The Scotch Yoke mechanism is for high-speed reciprocating motion. The 3-DoFs manipulator is composed of three stepper motors and they are mutually perpendicular. **b,** Magnetic field actuation of multi-legged millirobot with flexible tapered feet under discontinuous flap-wave (DFW) mode. As the magnetic bar is moved upper and forward, the robot raises its body corresponding to the front feet alignment to the magnetic flux. At the same time, the robot moves forward one step under the pulling force along the *y* axis. After the external magnetic field is off, all the robot's feet are in touch with the ground again. **c,** Magnetic field actuation of multi-legged millirobot with flexible tapered feet under continuous inverted-pendulum (CIP) mode. The magnetic bar is programmed to move to the left and right directions alternately, meanwhile maintaining a forward movement. In response to the magnetic flux, the robot exhibits a continuous locomotion-like human walking, characterized by the alternate rise-up and continuous forward motion. **d,** Magnetic field actuation of footless robot. In contrast, there is no any marked movement for the robot without the design of feet structure, even when the magnetic field is applied up to 200 mT.

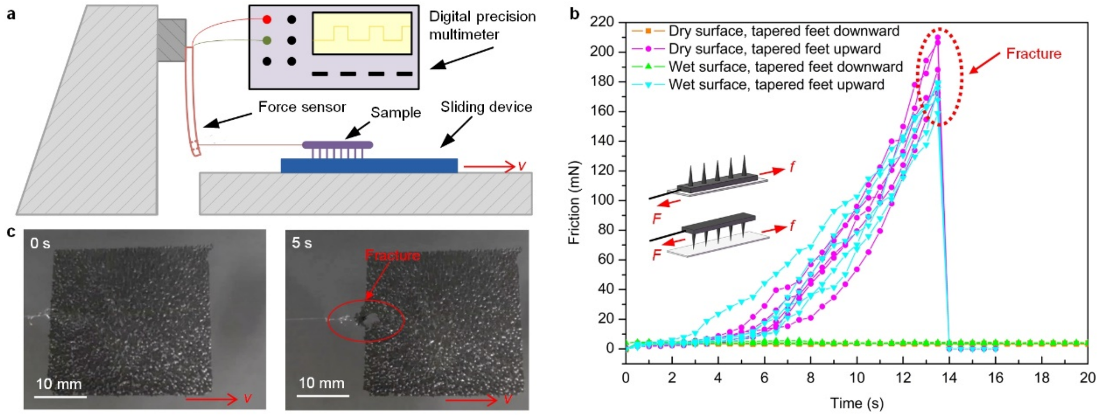

Supplementary Figure 5 | Friction measurement of the robot. a, Friction measurement experimental system set-up. During the friction experiment, square sample with the size of 30 mm × 30 mm is placed on the silicon wafer covered sliding device, which is driven with constant speed of 1.65 mm/s by stepper motor. **b,** Friction measurement experimental results. The friction force of robot with tapered feet contacting substrate is at least 40 times smaller than that without feet at both experiment conditions, i.e., dry condition (3.9 mN vs 188.2 mN), wet condition (4.1 mN vs 190.8 mN). **c,** Fractured point detection during the friction measurement. The labeled friction force for the footless robot in Extended Data Fig. 4b is the fracture force, the value of which is still less than the actual friction force (see Supplementary Methods).

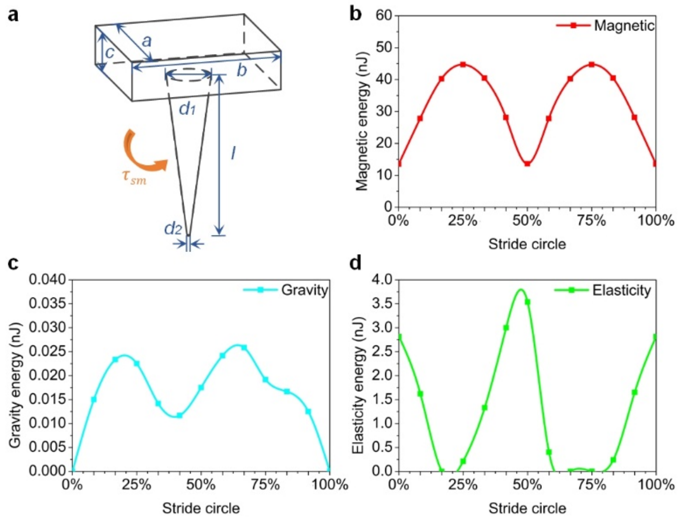

Supplementary Figure 6 | Single foot energy analysis in an individual gait cycle. a, Single flexible tapered foot model. To calculate the foot energy in an individual gait circle, several parameters are defined. **b,** Single flexible tapered foot magnetic energy variation. **c,** Single flexible tapered foot gravity energy variation. **d,** Single flexible tapered foot elastic energy variation. During the first 25% of the stride circle of single tapered foot, the stored elastic energy is released with the magnetic energy and gravity energy decreased. During the next 25% stride circle, the elastic energy transferred by magnetic energy and gravity energy is stored owing to the tapered foot soft property. After that, the robot body is lifted up during the elastic energy releasing process. Finally, the magnetic energy and gravity energy are transferred to elastic energy while the foot lands on the ground and backs to the initial state (see Supplementary Methods).

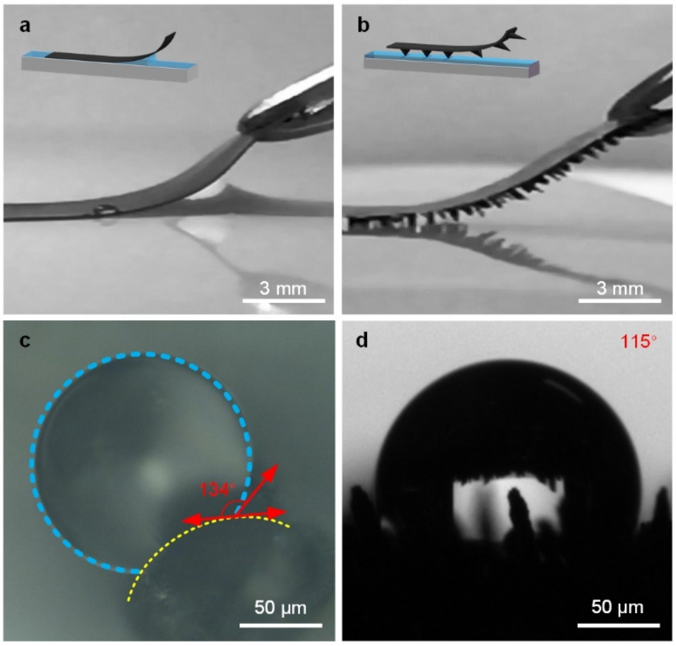

Supplementary Figure 7 | Robot measurement in humidity environment. a, Footless robot in contact with liquid membrane. **b,** Multiple tapered feet in contact with liquid membrane. **c,** Contact angle between droplet and robot leg. The contact angle is approximately 134°. **d,** Contact angle between droplet and multiple tapered feet robot. The contact angle is approximately 115°.

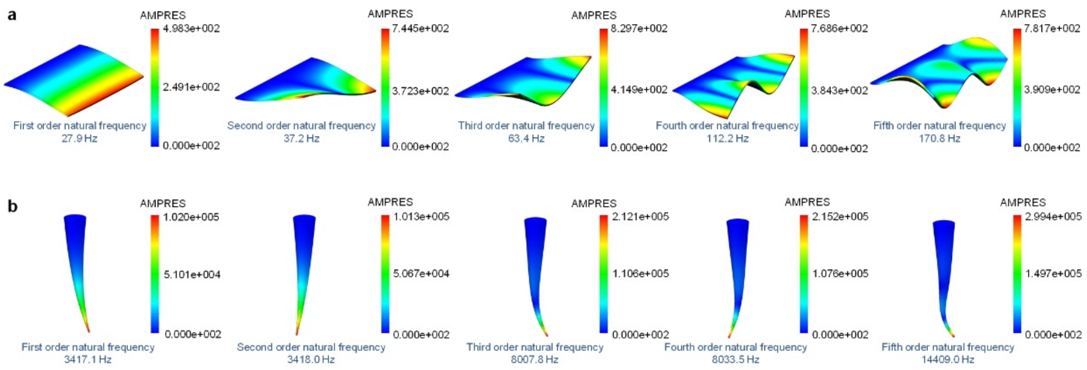

Supplementary Figure 8 | Natural frequency simulation. a, Natural frequency simulation of robot body. The millirobot body is simplified as a thin beam, and Euler-Bernoulli beam theory can be utilized to measure the natural frequency. **b,** Single tapered foot natural frequency simulation. The robot single tapered foot is modeled as a thick beam, and we utilize Timoshenko beam theory to measure the natural frequency (see Supplementary Methods).

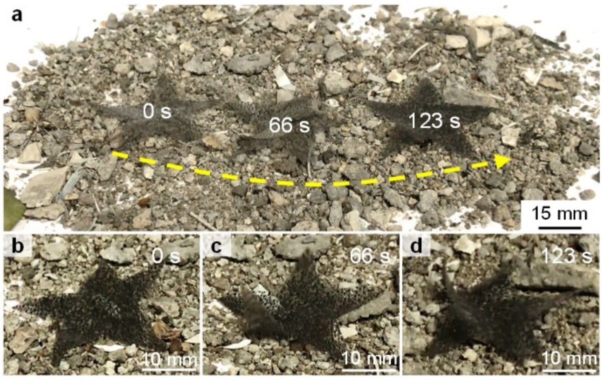

Supplementary Figure 9 | Bionic starfish millirobot locomotion demonstration. a, Actuated via external magnetic field, the starfish millirobot walks 10cm across the beach-like region in 123 s. **b, c and d,** Detailed postures of the bionic starfish multiple tapered feet millirobot during the motion. With the intricate regulation of the trajectory of the external magnetic field, the bionic starfish millirobot can manifest different postures (see Supplementary Methods). Via adjusting the posture of the moving robot, the robot is able to express similar motion characteristic with starfish (Supplementary Movie 9).

Supplementary Methods

1. Dimensionless Analysis L/S

The tapered feet (or leg) length to foot-to-foot spacing ratio (*L/S*) of typical bipedal animals are investigated, e.g., ostrich 4:1, human and crane 4:1, monkey 2.5:1, peacock 2:1, kangaroo and penguin 1.5:1, chicken, eagle and goose 1:1, duck 1:1.5. Typical four-leg animals: giraffe *L/S* 6:1, wolf 4:1, sheep 3.5:1, horse 3:1, bear, cow, koala, fox, hippo and elephant 2:1, panda, mouse, dog and pig 1.5:1, crocodile, rabbit, squirrel and cat 1:1, hedgehog 1:2. Typical six-leg animals: mosquito with *L/S* 7:1, ant 5:1, bee 2:1, fly, beetle and cockroach 1:1, cicada 1.5:1. Most of them located between 2:1 and 1:1. Typical eight-leg animals: octopus with *L/S* 3.5:1, spider 2.5:1, scorpion and lobster 2:1, crab 1:1. The *L/S* of typical soft animals: centipede 1:1, diplopod 1.5:1, pillworm 1:2.

As shown in Supplementary Figure 1, on the basis of extensive review of structural geometry of hundreds of ground animals, we find that the length of their legs (*L*) is normally 1~2 times larger than that of their foot-to-foot spacing (*S*). We hypothesis that it's a compromise between efficient locomotion and body supporting, since a large *L/S* is preferential for locomotion, but inferior to body supporting; to contrary, a small *L/S* is preferential for body supporting, but limited in locomotion. Interestingly, for animals with soft legs or feet such as starfish, centipede and pillworm, the *L/S* is typically approximately close to unity to make up the weakness of soft legs in body supporting. Therefore, in our robot design, we chose the *L/S* ≈ 1:1 for the multi-legged millirobot with flexible tapered feet

2. Robot Design and Manufacture

Although more and more soft robots have been applied in different domains, it is still challenging to achieve their performances (such as locomotion, loading, as well as obstacle-crossing ability) via surface structure using underlying materials. Especially in harsh liquid environments, the performances of robots are dramatically compromised due to the huge friction. Inspired by the polypod animals in nature, we design millirobots with multiple-legs to decrease the friction between the robots and harsh interfaces and increase the locomotion ability. Here we choose PDMS as the substrate of the robots due to its high biocompatibility, excellent elasticity and superhydrophobicity. One step magnetic particle-assisted molding approach in

this manuscript simplifies experimental process and avoids the use of templates. By controlling the frequency and direction of the external magnetic field, it is possible to achieve fast locomotion, excellent loading and obstacle crossing ability of the soft robots.

The multi-legged millirobot with flexible tapered feet was fabricated using modified magnetic particle-assisted molding approach. Briefly, we first prepare a mixture with Polydimethylsiloxane (PDMS) pre-polymer (containing 0.1 equivalent curing agents, purchased from Dow Corning, Sylgard 184), hexane, and magnetic particles (iron microparticles with an average diameter of 6-10 μm, purchased from Alfa Aesar). Here the weight ratio of the PDMS pre-polymer, magnetic particles and hexane (Sigma-Aldrich) is 2:1:1. Then, the mixture was well-distributed on the polystyrene plate through spin coating. After that, a permanent magnet with a size of 4 cm ×4 cm×2 cm was applied underneath the plate to form ordered micro tapered feet. The distance between the permanent magnet and the plate is 0.5 cm, corresponding to a magnetic intensity of 0.47 T. After the formation of the legs on the polystyrene plate, the plate was then placed in a convection oven for thermal curing for 1 h at 80 °C to solidify the PDMS (Supplementary Figure 2).

3. Single Permanent Magnet Model

$P\left(x_P, y_P, z_P\right)$ is an arbitrary point out of the rectangular permanent magnet and its total magnetic induction intensity **B** along the *x*, *y* and *z* direction can be represented as:

$$B_x = \frac{\mu_0 J_s}{8\pi}\Big[-\Gamma\left(0.5ma-x, 0.5mb-y, 0.5mc+z\right)-\Gamma\left(0.5ma-x, 0.5mb+y, 0.5mc+z\right) \\ +\Gamma\left(0.5ma+x, 0.5mb-y, 0.5mc+z\right)+\Gamma\left(0.5ma+x, 0.5mb+y, 0.5mc+z\right)\Big] \tag{1}$$

$$B_y = \frac{\mu_0 J_s}{8\pi}\Big[-\Gamma\left(0.5mb-y, 0.5ma-x, 0.5mc+z\right)-\Gamma\left(0.5mb-y, 0.5ma+x, 0.5mc+z\right) \\ +\Gamma\left(0.5mb+y, 0.5ma-x, 0.5mc+z\right)+\Gamma\left(0.5mb+y, 0.5ma+x, 0.5mc+z\right)\Big] \tag{2}$$

$$B_z = \frac{\mu_0 J_s}{4\pi}\Big[-\Psi\left(0.5mb-y, 0.5ma-x, 0.5mc+z\right)-\Psi\left(0.5mb+y, 0.5ma-x, 0.5mc+z\right) \\ -\Psi\left(0.5ma-x, 0.5mb-y, 0.5mc+z\right)-\Psi\left(0.5ma+x, 0.5mb-y, 0.5mc+z\right) \\ -\Psi\left(0.5mb-y, 0.5ma+x, 0.5mc+z\right)-\Psi\left(0.5mb+y, 0.5ma+x, 0.5mc+z\right) \\ -\Psi\left(0.5ma-x, 0.5mb+y, 0.5mc+z\right)-\Psi\left(0.5ma+x, 0.5mb+y, 0.5mc+z\right)\Big] \tag{3}$$

in which

$$\Gamma\left(\gamma_1, \gamma_2, \gamma_3\right) = \ln \frac{\sqrt{\gamma_1^2+\gamma_2^2+\left(\gamma_3-z_0\right)^2}-\gamma_2}{\sqrt{\gamma_1^2+\gamma_2^2+\left(\gamma_3-z_0\right)^2}+\gamma_2}\Bigg|_{z_0=0}^{z_0=mc} \tag{4}$$

$$\Psi\left(\psi_1,\psi_2,\psi_3\right)=\arctan\left[\frac{\psi_1\left(\psi_3-z_0\right)}{\psi_2\sqrt{\psi_1^{\ 2}+\psi_2^{\ 2}+\left(\psi_3-z_0\right)^2}}\right]\Bigg|_{z_0=0}^{z_0=mc} \tag{5}$$

Here, the permeability of vacuum μ_0 is $4\pi \times 10^{-7}$ H/m, J_s is the surface density of magnetizing current, and *ma, mb, mc* are the length, width, height of the rectangular permanent magnet respectively.

4. Magnetic Mechanism Analysis of Single Tapered Foot

When the multi-legged millirobot with flexible tapered feet is located in a magnetic field it would experience both a torque and a force. Both the magnetic torque and force the millirobot experienced are proportional to the magnetic field strength. The magnetic torque serves to align the millirobot with the magnetization field, e.g., raise the body; whereas the magnetic force serves as a driving force and drags the millirobot to move in the local maximum magnetic field.

Briefly, taking a single flexible tapered foot as a whole unit, the magnetic torque $\vec{T}_{sm}$ applied to the tapered foot can be calculated regardless of the foot's structure ("single chains" or "thick chains"). As illustrated in Supplementary Figure 6a, the magnetic torque applied to the tapered foot can be calculated as:

$$\vec{T}_{sm}=\int_{V_{sm}} d\vec{T}dV=\int_{V_{sm}}\left(\left(M_yB_z-M_zB_y\right)\vec{i}+\left(M_zB_x-M_xB_z\right)\vec{j}+\left(M_xB_y-M_yB_x\right)\vec{k}\right)dV \tag{6}$$

where $\vec{M}$ is the magnetization of the tapered foot, V_{sm} is the volume of the single flexible tapered foot and $\vec{B}(x,y,z)$ is the magnetic flux intensity. Suppose the angle between $\vec{M}$ and z axis is α, when the permanent magnet located below the robot moves forward along *y* axis, the magnetic torque along *x*, *y* and *z* axis can be represented as:

$$\begin{cases}\vec{T}_{smx}=B_z\sin\alpha\int_{V_{sm}}MdV-B_y\cos\alpha\int_{V_{sm}}MdV\\ \vec{T}_{smy}=B_x\cos\alpha\int_{V_{sm}}MdV\\ \vec{T}_{smz}=-B_x\sin\alpha\int_{V_{sm}}MdV\end{cases} \tag{7}$$

During the experiment, the permanent magnet is put underneath the robot with a distance of 20 mm, and then activated in the *y* direction to drive the robot forward. Here, the

permeability of vacuum μ_0 is $4\pi \times 10^{-7}$ H/m; the surface density of magnetizing current J_s is 3.1×10^4 A/m; the length, width and height of the rectangular permanent magnet are *ma*=40 mm, *mb*=40 mm, *mc*=20 mm respectively. The magnetization of the tapered foot $\vec{M}$ is measured experimentally by vibrating sample magnetometer (Cryogenic Inc.) (Supplementary Figure 6b). By substituting these values into Equations S1-S7, the theoretical magnetic torque applied to the tapered foot is successfully calculated, as the blue curve shown in Supplementary Figure 6c.

To verify the proposed theoretical model, we calculate the actual torque at various locomotion stage based on the leg's deformation by classical Euler-Bernoulli equation, and the results are also shown in Supplementary Figure 6c. The comparison result in Supplementary Figure 6c indicates that the theoretical value agrees well with the experimental value, which verifies the correction of the proposed magnetic driven model.

The magnetic drag force experienced by a single tapered foot can be represented as:

$$\vec{F}_{sm} = V_{sm} \cdot \left(\vec{M} \cdot \vec{\nabla}\right) \cdot \vec{B}(x, y, z) \tag{8}$$

Therefore, the magnetic drag force along *x*, *y* and *z* axis can be represented as:

$$\begin{cases} \vec{F}_{smx} = \left(\sin\alpha \dfrac{\partial B_y}{\partial x} + \cos\alpha \dfrac{\partial B_z}{\partial x}\right) \displaystyle\int_{V_{sm}} M dV \\ \vec{F}_{smy} = \left(\sin\alpha \dfrac{\partial B_y}{\partial y} + \cos\alpha \dfrac{\partial B_z}{\partial y}\right) \displaystyle\int_{V_{sm}} M dV \\ \vec{F}_{smz} = \left(\sin\alpha \dfrac{\partial B_y}{\partial z} + \cos\alpha \dfrac{\partial B_z}{\partial z}\right) \displaystyle\int_{V_{sm}} M dV \end{cases} \tag{9}$$

where V_{sm} is the volume of the single flexible tapered foot, $\vec{M}$ is the magnetization of the single flexible tapered feet, and $\vec{F}_{sm}$ is the force the single magnetic flexible tapered foot experiences. $\vec{B}(x, y, z)$ is magnetic flux intensity and the gradient in the field can be determined analytically by taking the derivatives of Supplementary Equations 1-5.

5. Friction Measurement

During the friction experiment, square sample with the size of 30 mm×30 mm is placed on the silicon wafer covered sliding device, which is driven with constant speed of 1.65mm/s

by stepper motor. The force sensor is connected to sample by string as exhibited in Supplementary Figure 4a. Four groups of experiments are carried out to test the corresponding friction in different conditions. In the wet contact surface condition, 3 ml deionized water is sprayed on 40 mm×180 mm wafer surface. The corresponding voltage output by force sensor is collected by multimeter (8846A Digital Precision Multimeter). The relationship between measured voltage V and force F is calibrated as equation:

$$F=(V-V_0)/k \tag{10}$$

where V_0 is the initial output voltage without force applied and scale factor k is equal to 0.0197.

As the results shown in Supplementary Figure 4b, the friction force of robot with tapered feet contacting substrate is at least 40 times smaller than that without feet at both experiment conditions, i.e., dry condition (3.9 mN vs 188.2 mN), wet condition (4.1 mN vs 190.8 mN). Note that the labeled friction force for the footless robot here is the fracture force (Supplementary Figure 4c), the value of which is still less than the actual friction force. What's more, the polylines of experiments groups with sample's feet downward, as shown as green and orange lines in Supplementary Figure 4b, are stable. Therefore, the multi flexible tapered feet can dramatically reduce the friction force and guarantee the soft robot to move smoothly on both dry and wet surface.

6. Discontinuous Flap-Wave Mode

To model the dynamics of the multi-legged millirobot with flexible tapered feet, we restrict modeling to a side-view of the robot in the x-z plane (Extended Data Fig. 6c). The robot dynamic relations are developed by:

$$m_f \ddot{y}_f = F_{yf} - F_f \tag{11}$$

$$m_f \ddot{z}_f = F_{zf} - m_f g \tag{12}$$

$$J\ddot{\theta}_f = T_{xf} + F_f \cdot r_f \cdot \sin\theta_f \tag{13}$$

where $\ddot{y}_f$ and $\ddot{z}_f$ represents the acceleration of the robot along y axis and z axis respectively. F_{yf} and F_{zf} represents the magnetic force that the robot experienced along y axis and z axis respectively. m_f is the robot mass and g is the acceleration of gravity. F_f represents sliding

friction force. J is the polar moment of inertia of the robot. $\ddot{\theta}_f$ and θ_f represents the robot orientation angle angular acceleration and angle respectively. r_f is the distance from robot's center of mass (COM) to the ground touching point.

7. Continuous Inverted-Pendulum Mode

Since the robot body is composed of single magnetic tapered foot, CIP movement cycle could be simplified as two single magnetic tapered feet structure moving forward alternatively. We establish the mathematic mode to analyze its motion and find that this kind of locomotion mode is very similar to that of human's inverted pendulum locomotion mode. When the supporting foot shifts into swing phase, the COM changes to the top of the other foot in support phase, so that this two single magnetic flexible tapered feet structure could be simplified into one inverted pendulum's movement. As illustrated in Supplementary Figure 6d, the robot's COM locates on the top of the two feet at the supporting phase, and then moves forward to the top of the swing foot when shifting into the swing phase. Such step repeats endlessly and finally results in the robot's continuous walking. Meanwhile, the whole robot body could be regarded as several two single magnetic flexible tapered feet structures, thus the robot's every movement could be simplified into multi inverted pendulums' movement (Supplementary Figure 6e). The equations of millirobot motion can be given by:

$$\ddot{y}_{mi} = \frac{F_{yi}}{m_i} \quad (14)$$

$$\ddot{z}_{mi} = \frac{F_{zi}}{m_i} - g \quad (15)$$

$$\dot{y}_{mi} = \int \ddot{y}_{mi}\, dt \quad (16)$$

$$\dot{z}_{mi} = \int \ddot{z}_{mi}\, dt \quad (17)$$

$$y_{mi} = \int \dot{y}_{mi}\, dt \quad (18)$$

$$z_{mi} = \int \dot{z}_{mi}\, dt \quad (19)$$

where F_{yi} and F_{zi} represents the magnetic field force the multiple tapered feet millirobot experienced along y axis and z axis respectively. m_i is the robot mass and g is the acceleration

of gravity. In addition, $\ddot{y}_{mi}$, $\dot{y}_{mi}$, y_{mi}, $\ddot{z}_{mi}$, $\dot{z}_{mi}$, z_{mi} represents the acceleration, velocity and displacement of the robot along *x* axis and *z* axis respectively.

And then, the robot foot landing position and the position of center of mass is $[y_{Fi} \quad z_{Fi}]$ and $[y_{mi} \quad z_{mi}]$.

The robot tapered foot length is given by:

$$L_i = \sqrt{\left(y_{Fi} - y_{mi}\right)^2 + \left(z_{Fi} - z_{mi}\right)^2} \tag{20}$$

and the robot tapered foot force is given by:

$$\begin{aligned} F_{Li} &= m_i g \cos\theta_i - m_i L_i \dot{\theta}_i^2 \\ &= m_i g \frac{z_{Fi} - z_{mi}}{L_i} - m_i g \frac{\dot{y}_{mi}{}^2 + \dot{z}_{mi}{}^2}{L_i} \end{aligned} \tag{21}$$

We add a damping to mimic the landing collision:

$$F_{di} = K_{di} \cdot \left(\dot{y}_{mi} \sin\theta_i + \dot{z}_{mi} \cos\theta_i\right) \tag{22}$$

$$F_{Li}{}' = F_{Li} + F_{di} \tag{23}$$

$$F_{yi} = F_{Li} \cdot \sin\theta_i \tag{24}$$

$$F_{xi} = F_{Li} \cdot \cos\theta_i \tag{25}$$

where F_{di} is damping force, K_{di} is damping coefficient and θ_i is the inverted pendulum initial angle.

8. Single Flexible Tapered Foot Analysis in an Individual Gait Cycle

A gait cycle is defined as beginning with the landing of one foot and ending at the next landing of the same foot. As illustrated in Fig. 3, the full gait cycle of an individual leg can be divided into four stages: Controlled Dorsiflexion (CD), Power Plantar Flexion (PPF), Swing State (SS) and Controlled Plantar Flexion (CPF) (Fig. 3a and Supplementary Movie 3). It can be seen that the motion of millirobot foot is very similar to the walking of human (Fig. 3b). Furthermore, the quantitative locomotion data of the tapered foot in one gait cycle is given in Fig. 3b, including the multi-legged millirobot with flexible tapered feet body’s displacement along *y* axis (pink square line), the foot’s displacement along *y* axis (green circle line), the robot

body's displacement along *z* axis (light blue regular triangle line) and the leg's deformation (dark blue inverted triangle line).

8.1 Controlled Dorsiflexion (CD)

In CD stage, with the change of magnetic field direction, the tapered foot begins at foot flat and continues veering until the foot has point contact with the ground, which can be deemed as reach the maximum dorsiflexion. In this stage, the stored elastic energy is released, which benefits the robot body movement along the *y* axis, as the pink square line depicted in Fig. 3b. Owing to the recovery of tapered foot deformation, the robot body is lifted up, as the light blue regular triangle line shown in Fig. 3b. The foot remains still during this stage, as the green circle line depicted in Fig. 3b.

8.2 Powered Plantar Flexion (PPF)

In PPF stage, with the change of magnetic field direction, additional elastic energy transferred by magnetic energy is stored owing to the tapered foot soft property, which leads to the body falling down as the light blue regular triangle line shown in Fig. 3b. The elastic energy storage can achieve high plantar flexion power in next SS stage. Meanwhile, the robot body continues moving along the *y* axis under the magnetic field drag force, as the pink square line depicted in Fig. 3b. The foot remains still during this stage, as the green circle line depicted in Fig. 3b.

8.3 Swing State (SS)

In SS stage, with the change of magnetic field direction, the tapered foot begins at toe-off and ends at step forward, realizing the stepping forward process, as the green circle line shown in Fig. 3b. Benefits from elastic energy stored in the PPF stage, the movement efficiency can be increased. Meanwhile, the robot body is lifted up along the *z* axis during the elastic energy releasing process and remains still along the *y* axis, as the light blue regular triangle line and pink square line depicts in Fig. 3b.

8.4 Controlled Plantar Flexion (CPF)

In CPF stage, with the recovery of the magnetic field direction, the foot lands on the ground and backs to the initial state. Both robot body and foot remain still along *y* axis during this stage, as the pink square and green circle lines shown in Fig. 3b. Under the effect of robot

gravity, the robot body falls down to store elastic energy in this stage, as the light blue regular triangle line shown in Fig. 3b.

9. Energy Analysis in an Individual Gait Cycle

9.1 Magnetic Energy

$P\left(x_P, y_P, z_P\right)$ is an arbitrary point out of out of the rectangular permanent magnet and its total magnetic energy density is shown as:

$$\omega_{magnetic} = \frac{B^2}{2\mu_0} \tag{26}$$

in which permeability of vacuum is $\mu_0 = 4\pi \times 10^{-7} H / m$

Magnetic energy can be represented as:

$$W_{magnetic} = \int_V \omega_{magnetic} dV \tag{27}$$

As depicted in Supplementary Methods section 3, any point $P\left(x_P, y_P, z_P\right)$ out of the rectangular permanent magnet total magnetic induction intensity *B* along the *x*, *y* and *z* direction on the component is represented in equations S1-S5.

As shown in Supplementary Figure 7a, single flexible tapered foot is composed of the body and the foot. The body is thin film with side length $a=b=600$ μm and thickness $c=150$ μm. The foot is with length $l=650$μm, root circle diameter $d_1=150$ μm and the tip circle diameter $d_2=10$ μm, so that the volume of the single tapered foot is:

$$V_{leg} = abc + \frac{1}{3}\pi l\left(\left(\frac{1}{2}d_1\right)^2 + \left(\frac{1}{2}d_2\right)^2 + \frac{1}{4}d_1 d_2\right) \tag{28}$$

By calculating Supplementary Equations 26-28, the magnetic energy variation of the single tapered foot during one circle is shown in Supplementary Figure 7b.

9.2 Gravitational Potential Energy

The gravitational potential energy E_P of the single flexible tapered foot can be represented as:

$$E_P = mgh \tag{29}$$

where *m* is the mass of single flexible tapered foot, *g* is gravitational acceleration and *h* is the foot height.

Through analyzing the single flexible foot barycenter variation, the gravitational potential energy variation of the single foot during one circle can be calculated as shown in Supplementary Figure 7c.

9.3 Elastic Energy

We define that the single flexible tapered foot is subject to a torque load τ_{sm}, as shown in Supplementary Figure 7a, the elastic energy of the single foot can be represented as:

$$V_{\varepsilon} = \int_{l}^{0} \frac{1}{2} \tau_{sm} d\left(\theta_{sm}\right) \quad (30)$$

in which θ_{sm} is the single foot angle of rotation.

$$\omega\left(x\right) = \frac{\tau_{sm} l}{EI} \quad (31)$$

in which E = 2 MPa, represented as Young's modulus.

By substituting Supplementary Equation 31 into Supplementary Equation 30, the elastic energy variation of the single foot during one circle can be calculated as shown in Supplementary Figure 7d.

During the first 25% of the stride circle of single tapered foot, the stored elastic energy is released with the magnetic energy and gravity energy decreased. During the next 25% stride circle, the elastic energy transferred by magnetic energy and gravity energy is stored owing to the tapered foot soft property. After that, the robot body is lifted up during the elastic energy releasing process. Finally, the magnetic energy and gravity energy are transferred to elastic energy while the foot lands on the ground and backs to the initial state.

10. Natural Frequency Analysis

To estimate the limiting speed of the multi-legged millirobot with flexible tapered feet, the natural frequency of the millirobot body and flexible tapered foot is analyzed.

10.1 Body natural frequency

As shown in Supplementary Figure 8a, the millirobot body is simplified as a thin beam, so that Euler-Bernoulli beam theory can be utilized to measure the natural frequency.

The motion differential equation of cantilever beam is:

$$EI \frac{\partial^4 w(x,t)}{\partial x^4} + \rho A \frac{\partial^2 w(x,t)}{\partial t^2} = 0 \quad (32)$$

The boundary condition of the cantilever beam is:

$$w(x=0)=0(1), \frac{dw}{dx}(x=0)=0(2), \left.\frac{\partial^2 w}{\partial x^2}\right|_{x=l}=0(3), \frac{\partial}{\partial x}\left(EI\frac{\partial^2 w}{\partial^2 x}\right)\Bigg|_{x=l}=0(4); \tag{33}$$

The free vibration of this partial differential equation is obtained:

$$w(x,t)=W(x)T(t) \tag{34}$$

By calculating Supplementary Equations 32-34, the first order of this robot body natural frequency is:

$$\omega_1=1.875104^2\left(\frac{EI}{\rho Al^4}\right)^{\frac{1}{2}}\approx 22.571Hz \tag{35}$$

which is almost in accordance with the simulation results 27.889Hz, as shown in Supplementary Figure 8a.

10.2 Single leg natural frequency analysis

As shown in Supplementary Figure 8b, the robot single tapered foot is modeled as a thick beam, thereby Timoshenko beam theory can be utilized to measure the natural frequency.

The free vibration differential equation of cantilever beam is:

$$EI\frac{\partial^4 w(x,t)}{\partial x^4}+\rho A\frac{\partial^2 w(x,t)}{\partial t^2}-\rho I\left(1+\frac{E}{kG}\right)\frac{\partial^4 w}{\partial x^2\partial t^2}+\frac{\rho^2 I}{kG}\frac{\partial^4 w}{\partial t^4}=0 \tag{36}$$

Boundary conditions:

$$w(x=0)=\phi(x=0)=0(1), \left.\frac{\partial w}{\partial x}-\phi\right|_{x=l}=\left.\frac{\partial \phi}{\partial x}\right|_{x=l}=0(2) \tag{37}$$

The general solution to the equation is:

$$w(x,t)=C\sin\frac{n\pi x}{l}\cos w_n t \tag{38}$$

By calculating Supplementary Equations 36-38, the first order of this robot tapered foot natural frequency is:

$$w_1=\sqrt{\frac{EI}{\rho A}}\frac{\pi^2}{l^2}\approx 3.234kHz \tag{39}$$

which is almost in accordance with the simulation results 3.417kHz, as shown in Supplementary Figure 8b.

11. Bionic Starfish Millirobot

Inspired by the morphology and tube feet of starfish, we design the bionic starfish multiple flexible tapered feet millirobot. Here, we choose five-rayed starfish robot prototype as an example to demonstrate the feasibility of our multiple flexible tapered feet millirobot, as shown in Supplementary Figure 9. The starfish millirobot is a pentagram with side length 10mm and its weight is only 46.5mg. We spray sand mixed with tiny stone and dry leaves on a piece of white sheet to mimic the beach environment that real starfish walks. Actuated via external magnetic field, the starfish millirobot walks 10 cm across the beach-like region in 123s, as shown in Supplementary Figure 9a and Supplementary Movie 9. Detailed postures of the bionic starfish multiple tapered feet mil
