# Supplementary material for: A bioinspired multilegged soft millirobot that functions in both dry and wet conditions
Source: Nat Commun. 2018 Sep 26;9:3944. doi: 10.1038/s41467-018-06491-9 (PMC6158235; doi:10.1038/s41467-018-06491-9)
Supplement: Supplementary file 3 — Description of Additional Supplementary Files [file 41467_2018_6491_MOESM3_ESM.pdf]

File Name: Supplementary Movie 1

Description: Two basic types of motion modes. The left video shows the multi-legged millirobot with flexible tapered feet locomotion with discontinuous flap-wave mode (DFW). This motion mode is generated by an "O" trajectory of magnet with surface magnetic field intensity 80 mT on *y*-*z* plane. This multi-legged millirobot with flexible tapered feet moves 8 mm in 36 s with frequency 1 Hz. The right video shows the multi-legged millirobot with flexible tapered feet locomotion with continuous inverted-pendulum mode (CIP). This motion mode is generated by an "S" trajectory of magnet with surface magnetic field intensity 80 mT on *x*-*y* plane. This multi-legged millirobot with flexible tapered feet moves 12 mm in 20 s with frequency 1 Hz. The video is at 1× playback speed.

File Name: Supplementary Movie 2

Description: An individual foot motion in one gait circle. The video shows the continuous motion of an individual foot in each gait cycle with frequency 1 Hz, as the results given in Fig. 3a and Fig. 3b, including the robot body's displacement along *y* axis (pink line), the foot's displacement along *y* axis (green line), the robot body's displacement along *z* axis (light blue line) and the foot's deformation (dark blue line). The video is at 0.1× playback speed.

File Name: Supplementary Movie 3

Description: Over-obstacle ability comparison between the flexible foot and the rigid foot. The video shows when multi-legged millirobot with flexible tapered feet comes to an obstacle (height 160 μm × width 1 mm) on the way forward, the robot could stride it via foot deformation after head lifting with an angle approximate 20° by changing the magnetic flux direction. By contrast, the robot with rigid foot gets stuck to the obstacle no matter how magnetic flux direction changes.

File Name: Supplementary Movie 4

Description: Robot locomotion on liquid film. The video shows the difference when footless robot and multiple tapered feet robot contact to liquid membrane. The video is at 4× playback speed. When the multiple tapered feet millirobot occurred to a humidity environment (liquid film approximate 150 μm), it moves 10 mm with an average speed of 0.5 mm/s with frequency 1 Hz. The video is at 1× playback speed.

File Name: Supplementary Movie 5

Description: Robot locomotion with a loading approximate 100 times of its own weight. The video shows the multi-legged millirobot with flexible tapered feet motion when it is loaded a capsule weight 3980.6 mg with self-weighted 39.4 mg, moves 8 mm in 45.3 s with frequency 1 Hz. The video is at 8× playback speed.

File Name: Supplementary Movie 6

Description: Robot cross an obstacle with height approximate 10 times higher of its own foot. The video shows the multi-legged millirobot with flexible tapered feet climbs two obstacles with height 6.5 mm, and slop angle 60° in 113 s within 40 mm distance. The beginning of the video emphasizes the “soft” property. The video is at 8× playback speed.

File Name: Supplementary Movie 7

Description: Robot motion demonstration with swing frequency at 0.5 Hz, 4 Hz and 16 Hz. The video shows the multi-legged millirobot with flexible tapered feet is actuated by a permanent magnet with surface magnetic field intensity 200 mT at frequency 0.5 Hz, 4 Hz and 16 Hz. The robot moves with velocity around 0.98 mm/s at 0.5 Hz, 8.3 mm/s at 4 Hz frequency and 25.2 mm/s at 16 Hz frequency.

File Name: Supplementary Movie 8

Description: Robot demonstration of locomotion ability in harsh environment. The video shows the complex stomach model’s internal structure and humidity environment, which demonstrates the multi-legged millirobot with flexible tapered feet potential locomotion ability in-vivo environment. The video is at 4× playback speed.

File Name: Supplementary Movie 9

Description: Bionic starfish millirobot locomotion demonstration. The video shows the beach environment with sand, tiny stone and dry leaves on a piece of white sheet. The starfish millirobot walks 10 cm across the beach-like region in 123 s. The video is at 8× playback speed.
